# Citrinin Mycotoxin Contamination in Food and Feed: Impact on Agriculture, Human Health, and Detection and Management Strategies

**DOI:** 10.3390/toxins14020085

**Published:** 2022-01-23

**Authors:** Madhu Kamle, Dipendra Kumar Mahato, Akansha Gupta, Shikha Pandhi, Nitya Sharma, Bharti Sharma, Sadhna Mishra, Shalini Arora, Raman Selvakumar, Vivek Saurabh, Jyoti Dhakane-Lad, Manoj Kumar, Sreejani Barua, Arvind Kumar, Shirani Gamlath, Pradeep Kumar

**Affiliations:** 1 Applied Microbiology Laboratory, Department of Forestry, North Eastern Regional Institute of Science and Technology, Nirjuli 791109, India; madhu.kamle18@gmail.com; 2 CASS Food Research Centre, School of Exercise and Nutrition Sciences, Deakin University, Burwood 3125, Australia; kumar.dipendra2@gmail.com (D.K.M.); shirani.gamlath@deakin.edu.au (S.G.); 3 Department of Dairy Science and Food Technology, Institute of Agricultural Sciences, Banaras Hindu University, Varanasi 221005, India; salonigupta.ag@gmail.com (A.G.); shikhapandhi94@gmail.com (S.P.); sbharti51997@gmail.com (B.S.); sadhnamishra2649@gmail.com (S.M.); arvind00000@gmail.com (A.K.); 4 Food Customization Research Laboratory, Centre for Rural Development and Technology, Indian Institute of Technology Delhi, New Delhi 110016, India; nitya.sharma64@gmail.com; 5 Faculty of Agricultural Sciences, GLA University, Mathura 281406, India; 6 Department of Dairy Technology, College of Dairy Science and Technology, Lala Lajpat Rai University of Veterinary and Animal Sciences, Hisar 125004, India; shaliniarora.luvas@gmail.com; 7 Centre for Protected Cultivation Technology, ICAR-Indian Agricultural Research Institute, Pusa Campus, New Delhi 110012, India; selvakumarsingai@gmail.com; 8 Division of Food Science and Post-Harvest Technology, ICAR-Indian Agricultural Research Institute, New Delhi 110012, India; vivek.bhu12@gmail.com; 9 Technology Transfer Division, ICAR-Central Institute for Research on Cotton Technology, Mumbai 400019, India; jyotip.dhakane@gmail.com; 10 Chemical and Biochemical Processing Division, ICAR—Central Institute for Research on Cotton Technology, Mumbai 400019, India; manoj.kumar13@icar.gov.in; 11 Department of Agricultural and Food Engineering, Indian Institute of Technology, Kharagpur 721302, India; sreejani301@iitkgp.ac.in; 12 Max Planck Institute for Polymer Research, Ackermannweg 10, 55128 Mainz, Germany

**Keywords:** citrinin, food and feed contamination, human health, detection and management strategies

## Abstract

Citrinin (CIT) is a mycotoxin produced by different species of *Aspergillus*, *Penicillium*, and *Monascus*. CIT can contaminate a wide range of foods and feeds at any time during the pre-harvest, harvest, and post-harvest stages. CIT can be usually found in beans, fruits, fruit and vegetable juices, herbs and spices, and dairy products, as well as red mold rice. CIT exerts nephrotoxic and genotoxic effects in both humans and animals, thereby raising concerns regarding the consumption of CIT-contaminated food and feed. Hence, to minimize the risk of CIT contamination in food and feed, understanding the incidence of CIT occurrence, its sources, and biosynthetic pathways could assist in the effective implementation of detection and mitigation measures. Therefore, this review aims to shed light on sources of CIT, its prevalence in food and feed, biosynthetic pathways, and genes involved, with a major focus on detection and management strategies to ensure the safety and security of food and feed.

## 1. Introduction

Mycotoxins are poisonous secondary metabolites produced by filamentous fungi infesting crops and grain before harvest in the field or after harvest during storage [1]. Improper storage conditions regarding moisture, temperature, and water activity play a significant role in the proliferation of storage fungi and the production of toxins. Citrinin (CIT) is a polyketide-derived mycotoxin most commonly occurring during storage [2,3,4]. Hetherington and Raistrick isolated CIT for the first time from a culture of *Penicillium citrinum* in 1930s [5]. Meanwhile, it was reported that the three fungal genera *Penicillium* (*P. citrinum*, *P. verrucosum*, and *P. expansum*), *Aspergillus* (*A. carneus*, *A. niveus*, *and A. terreus*), and *Monascus* (*M. ruber*) could produce CIT [4,5,6,7]. Recently, CIT has also been found in food colorings traditionally made in Asia from rice fermented with *Monascus purpureus* (“red mold rice”), conventionally used for meat preservation and food coloring [4,8].

Mycotoxins can contaminate the final food products and pose health concerns. However, recent advancements in food processing, such as hazard analysis of critical control points (HACCP) and good manufacturing practices (GMP), have aided in keeping final food products safe and healthy [9,10]. Apart from this, several degradation methods can be applied for the partial or complete elimination of these toxins from food to ensure consumer food safety and avoid health concerns. Though CIT has shown antibacterial [11], anticancer [12], and neuroprotective [13] properties, it is seldom used as a drug owing to its high nephrotoxicity and genotoxicity. Various in vitro and in vivo studies provided strong evidence of reproductive toxicity as well as the teratogenic and embryotoxic effects of CIT [4,14]. However, the International Agency for Research on Cancer (IARC) has classified CIT in Group III of carcinogens due to limited evidence in experimental animals and no evidence in humans regarding its carcinogenicity [4].

This review overviews the sources, chemistry, and biosynthesis of CIT, the effects of environmental factors on CIT production, its occurrence in food and feed, and the health effects in humans and animals. Moreover, the effects of processing techniques on CIT and various degradation methods with a major focus on detection and management strategies in food and feed are discussed.

## 2. Major Source of Citrinin

The fungi of the genera *Penicillium*, *Aspergillus*, and *Monascus* are major producers of CIT [4,5]. *Penicillium* spp. are of foremost importance and are reported to produce CIT worldwide during the drying and storage of cereal crops and other foodstuffs [14], among which *Penicillium citrinum* occurs most commonly in all kinds of food and feed, in almost all climatic conditions. Table 1 provides an overview of the current identity of microfungi *Penicillium*, *Aspergillus*, and *Monascus* species that can apparently produce CIT in foodstuffs [4,6,7,15,16,17].

## 3. Chemistry and Biosynthesis of Citrinin

**Figure 1:**
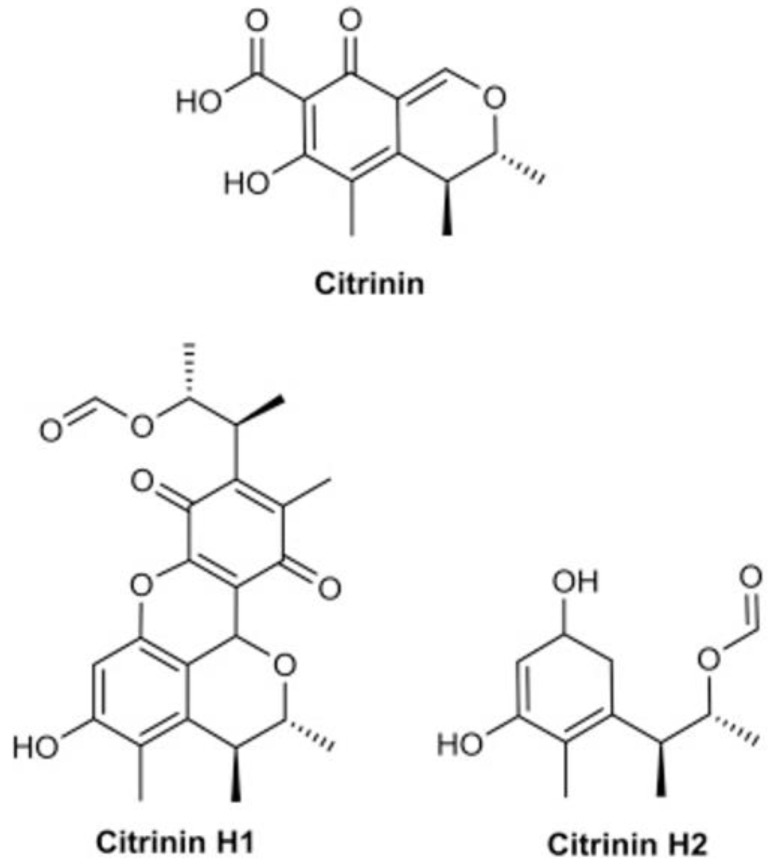
Chemical structures of citrinin and its decomposition products citrinin H1 and citrinin H2.

CIT (Figure 1) is a polyketide-containing mycotoxin (C_13_H_14_O_5_, IUPAC: (3*R*, 4*S*)-4,6-dihydro-8-hydroxy3,4,5-trimethyl-6-oxo-3*H*-2-benzopyran-7-carboxylic acid). It is a solid poison with the appearance of lemon-yellow needles at pH 4.6. The color changes to cherry red at pH 9.9. It melts at temperatures between 175 and 178.5 °C. In practice, it is insoluble in cold water but somewhat soluble in hot water, and it is soluble in aqueous sodium hydroxide, sodium carbonate, or sodium acetate, as well as in polar organic solvents such as ethanol, methanol, and acetonitrile. It has a UV-light absorption maximum ranging from 250 to 321 nm depending on the solvent [18]. CIT decomposes at temperatures exceeding 175 °C in dry conditions and over 100 °C in wet ones [19]. CIT can be destroyed by acidic or alkaline liquids, as well as by heating. CIT is a quinone with two intramolecular hydrogen bonds [14].

**Figure 2:**
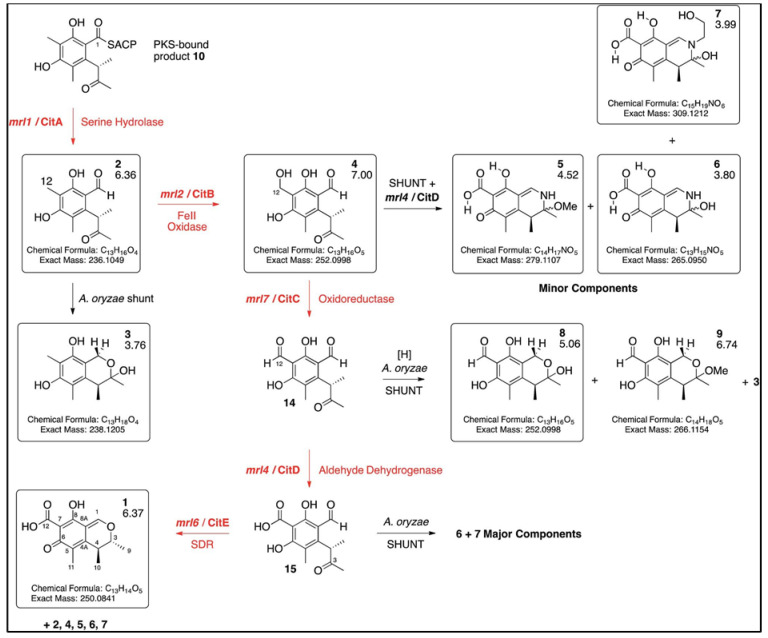
Schematic illustration of biosynthesis of CIT (adopted from He and Cox [5]).

CIT is synthesized via the polyketide pathway [20]. It is generally known that three secondary metabolites, pigments, monacolin K, and citrinin, share a biochemical route before a specific branch step, which is generated by acetyl CoA and malonyl-CoA, particularly for pigment and citrinin. More research is needed to determine the impact of disrupting the biosynthetic pathway on the production of pigments and citrinin [21]. CIT biosynthesis in the genus *Monascus* appeared to be strain-specific. The compound biosynthesis appears to be a tetraketide rather than a pentaketide, as found in *Aspergillus terreus* and *Penicillium citrinum*. The tetraketide produced in *Monascus* is created by the condensation of one acetyl-CoA molecule with three malonyl-CoA molecules [22]. He and Cox [5] established the biosynthetic route of CIT in *M. ruber* M7 via target gene deletion and heterologous expression in *Aspergillus oryzae*, which involved the creation of an unreduced trimethyl pentaketide (thioester) by CitS, a non-reducing polyketide synthase (nrpks). The discovery of keto-aldehyde as the first enzyme-free intermediate with nrpks appears to be aided by a cryptic CitA-catalyzed hydrolysis step. CitB is a non-heme iron oxidase that catalyzes the conversion of methyl to alcohol. CitC catalyzed the conversion of alcohols to aldehydes, whereas CitD catalyzed the conversion of aldehydes to carboxylic acids. CitE catalyzed the end catalysis of C-3, resulting in CIT production (Figure 2) [5,23].

## 4. Genes Responsible for Citrinin Production

Most polyketide metabolite pathway genes are grouped together. Six of the genes have comparable sequences to *Monascus purpureus* BCRC33325 citrinin biosynthesis pathway genes. *PksCT*, *ctnA*, *orf1*, *orf3*, *orf4*, and *orf5* are all implicated. They successively encode a polyketide synthetase, a regulator, an aldehyde dehydrogenase, an oxygenase, an oxidoreductase, and a membrane transporter [20,24]. The capacity to generate citrinin was lost in the *pksCT* disruptant. The *pksCT* mutant was not genetically stable, and citrinin production was recovered following repeated culture [24]. Similarly, in *Monascus aurantiacus*, loss of *pksCT* resulted in a significant reduction in citrinin synthesis. Surprisingly, this mutant was able to generate more red and yellow pigments [25]. Furthermore, transcriptional regulation of fungal secondary metabolite pathways is tightly controlled. In *M. purpureus*, *ctnA* is a key transcriptional activator of citrinin production. The deletion of *ctnA* dramatically reduced the production of the *pksCT* transcript, which resulted in lower citrinin production [26,27]. The phenotype of citrinin production is lost when *pksCT* is disturbed. The *ctnA* gene encodes a Zn(II)2Cys6 binuclear DNA binding protein that is a significant inducer of citrinin synthesis. As a result, the *ctnA*-deficient strain of *M. purpureus* produces so little CIT that it is barely detectable [22].

## 5. Occurrence in Food and Feed

CIT is found mainly in foodstuffs of vegetable origin. In addition, its presence is detected in various cereals (maize, wheat, rye, rice, corn, barley, oat) and cereal-based products, pomaceous fruits and fruit juices, roasted nuts (almonds, peanuts, hazelnuts, pistachio nuts), oilseeds (e.g., sunflower), and spices (e.g., turmeric, coriander, fennel, black pepper, cardamom and cumin) [15,16,28,29]. Cheese is also contaminated by CIT where toxigenic strains directly grow in the cheese mass [30]. CIT production is most likely when grains are not properly dried, retaining higher moisture content (>16%). The favorable temperature range for growth of CIT is between 12 and 37 °C, with an optimum temperature at 30 °C [1,4,17]. Data on the occurrence of CIT in food and feed around the world are listed in Table 2.

## 6. Effects on Agricultural Food and Feed

Agricultural products can be contaminated during pre-harvest, harvest, and post-harvest conditions. CIT contaminates harvested grains, dairy products, spices, juices from fruits and vegetables, herbs, and citrus fruits [23]. Wang et al. [85] observed the significant presence of toxigenic strains of CIT in cheese. According to the EFSA [28], CIT is mostly found in a few agricultural products, fruits, biological fluids, animal feed, and dairy products. Irradiation (ultraviolet, UV) of fruits containing 280–400 μg/kg of CIT resulted in destruction, with no presence of CIT found in the fruits. Following the report by the EFSA [28], CIT can be associated with ochratoxin A and aflatoxin B in grain products and cereals as well as with patulin in the case of apple juices and apple jams [28]. CIT production is also regulated by nutritive elements such as oxygen availability, fatty acids, nitrogen, and carbon sources, besides environmental factors such as water activity, temperature, commodity preservation, and storage conditions [85]. There is a regular occurrence of CIT in food and feed with the potential chance of consumer exposure to the toxin; still, no legal limits have been set [86]. However, the European Commission declared the presence of a maximum safe limit of up to 100 µg/kg in food supplements [87]. CIT in fermented red rice has a maximum limit of 50 µg/kg in China and 200 µg/kg in Japan [75]. In addition, the European Union in 2014 has set a maximum limit of 2000 µg/kg in rice fermented with red yeast *M. purpureus* [88].

## 7. Mechanism of Toxicity and Health Effects of Citrinin

### 7.1. Mechanism of Toxicity

The two basic mechanisms of CIT-mediated harmful effects in biological systems are assumed to be the effects of oxidative stress and altered enzymatic antioxidative responses (e.g., epithelial glutathione and transhydrogenase) [14]. In the respiratory chain, CIT has been discovered to promote the creation of reactive oxygen species (ROS) and boost the synthesis of superoxide anions. These bioactivities could explain lipid peroxidation [89] and cell death associated with mitochondrial malfunction [90]. The activation of caspases-3, -6, -7, and -9 has been linked to CIT triggered apoptosis in kidney PK15 cells and human promyelocytic leukemia (HL-60) cells [91]. CIT (108, 324, and 970 ppm) has been shown in several studies to cause harmful consequences in varieties of yeast cells by inducing oxidative stress and upregulating genes from oxidative stress response such as AADs, OYE3, FLR1, GRE2, and MET17 [92,93].

CIT has previously been shown to accumulate in the budding yeast mitochondria, and exposure with CIT causes malfunction of respiratory system as well as mitochondrial complex I inhibition [94]. Dysfunction in mitochondria caused by suppression of mitochondrial complex I resulted in superoxide anion (O_2_^−^) production. Similarly, exposure with CIT for 60 min increased the amount of ROS in hepatocarcinoma HepG2 cells (10–30 μM) [95]. As a result, it seems that CIT-induced ROS generation is required for initiation in apoptosis and that antioxidant system activation and adaptive responses mediated through ROS-sensitive transcription factors are activated. CIT treatment (1000 µM) of cells (107 mL^−1^) for 60 min at pH 4.5 resulted in a considerable rise in peroxides and total ROS as well as a 3-fold increase in glutathione concentration, with no change in superoxide or hydroxyl radical levels [96]. CIT treatment raised ROS levels in hepatocarcinoma HepG2 cells (10–30 µM) for 60 min [95] and in single cells from the murine skin suspensions at 50 µM for 12–72 h [97]. This suggests that CIT-induced ROS generation is required for apoptosis and antioxidant system activation, as well as for adaptive responses, which are mediated through the activation of ROS-sensitive transcription factors. A decrease in GSH due to conjugation with patulin as well as molecular interactions of CIT with the free sulfhydryl groups of integrative membrane proteins lead to cell death [96]. CIT may affect the plasma membrane by inhibiting 3-hydroxy-3-methylglutaryl-coenzyme in a time-dependent, irreversible manner. Long-term exposure to a reductase disrupts the synthesis of the cholesterol/ testosterone (and ergosterol) pathway, resulting in hypocholesterolemia [98]. In acute testing, CIT inhibited growth, cell proliferation, viability, cytotoxicity, and many other measured parameters in a dose- and time-dependent manner, regardless of the kind of cells used [96]. In chronic tests, CIT I inhibited one of the key enzymes involved in cholesterol synthesis (resulting in lower serum testosterone levels and hypocholesterolemia) [99], had multiple immune modulator effects [100], and caused nephropathy, hepato- and fetotoxicity, and renal adenoma formation in various animal models [101].

### 7.2. Health Effects of Citrinin

CIT has been shown to be nephrotoxic and hepatotoxic to humans. The kidney is the major target organ of CIT [9]. CIT is commonly found along with ochratoxin, and an additive or synergic effect has been shown to increase the toxicity, causing kidney disease in humans [102,103]. Other than the kidney, the target organs of CIT include the liver, mitochondrial respiratory chain, and bone marrow [14]. This nephrotoxin is also considered as one possible reason for porcine nephropathy. In the absence of adequate exposure data, the risk of CIT as a food contaminant was assessed based on an estimate of the critical CIT concentrations in grains and grain-based products that would result in nephrotoxicity [28]. Furthermore, CIT is quickly absorbed and transported, particularly to the liver and kidney. A recent human CIT toxic kinetic study revealed that 40% of CIT was eliminated in the urine, implying that 40% of CIT was absorbed [17]. In a study by López Sáncheza, et al. [104], CIT in European foodstuffs was identified, where red yeast rice (RYR) samples (*n* = 92) were reported to be highly susceptible to CIT contamination. The findings of this study demonstrate the uncertainty regarding the carcinogenicity and genotoxicity of CIT, leading to a reconsideration of selection of the optimum level to protect public health and particularly consumers of RYR supplements [87].

Furthermore, CIT, when examined at 20 and 50 μg in embryos of Zebrafish, reduced blood flow rate and heartbeat, probably through modulating the expression of the jun B and tbx2a genes. Male and female mice subjected to CIT therapy at 0.065 mg/100 g (p.o.) for 60 days showed morphological changes as well as a noteworthy reduction in weight in the body and organs. Hemosiderin granules were found in some of the organs as well. Male infertility was discovered, as well as changes in parameters of hematology [105]. In mouse blastocysts, CIT at 15 or 30 µM has been shown to enhance apoptotic events and decrease overall cell counts. CIT (2.5 and 5.0 µM) has also been observed to impair the maturation rate of the oocyte, as well as fertilization and embryo development in ICR mice [106]. In Kunming strain male mice (*n =* 6), CIT at 0–6.25 mg/kg body wt./d (i.p.) for 7 days resulted in various substantial alterations which included an increase in the epididymis, the relative weights of the testes, preputial glands, and seminal vesicles, and the quantity of aberrant spermatozoa. Furthermore, when CIT-exposed males were mated with females, the pregnancy rate was shown to be lower, implying a negative influence on the reproductive system. In research conducted with 40 mg/kg body wt./d CIT (gastric intubation) in 14-day-old male and female guinea pigs (*n =* 5 to 15), Thacker et al. [107] observed several alterations including dehydration, loss of body weight, liver and kidney damage (primarily degeneration and necrosis), and anemia. Jordan et al. [108] found that CIT at 62 and 87 mg/kg body wt. (p.o.) caused kidney lesions in female mice (*n =* 5 to 15), with renal tubular dilatation and deposition of protein within the lumen of the renal tubules. Furthermore, in rabbits (*n =* 6), Hanika et al. [109] observed that CIT (33.5 or 77 mg/kg body wt./d administered for 7 days) caused renal changes including minor tubular regeneration and necrosis, as well as tubular degeneration. Jordan et al. [110] reported that only one intraperitoneal (i.p.) dose of CIT (50 mg/kg body wt.) could cause nephrosis in Sprague–Dawley rats, based on increased blood and glucose in urine within the initial 3 hours. CIT administered at a dosage of 120 mg/kg body wt. (p.o.) caused metabolic acidosis and azotaemia as well as hypokalemia and hemoconcentration within 4–12 h in male New Zealand white rabbits [111].

## 8. Effects of Processing on Citrinin

The toxigenic potential of the fungi and thereby the yield of toxins can be affected by conditions during harvest, storage, and processing operations. CIT levels decreased in the products after processing due to its sensitivity to heat. CIT is decomposed into two other complexes, namely CIT-H1 and CIT-H2, after the heat treatment generally above 175 °C under dry conditions and above 100 °C in the presence of water [101,112]. CIT-H2 has lower toxicity than CIT, while CIT-H1 is more toxic [101,113]. Dicitrinin A is another decomposition product of CIT reported recently together with other degradation products [17,28,114]. Besides temperature, the addition of compounds such as flavonoids can also affect the level of CIT. Wang et al. [115] investigated the effect of isoflavone and genistein on CIT production by *Monascus aurantiacus* Li AS3.4384 (MAL) during liquid-state fermentation containing rice powder as a carbon source and 2.0 g/L genistein. The results showed a significant reduction in CIT levels (approximately 80%) and an increase in biomass. Other flavonoids such as quercetin, kaempferol, myricetin, and genistin were also tested for their effectiveness; however, the maximum reduction was observed in the case of genistein. Further research by Ouyang et al. [116] showed that the reduction was due to changes occurring at the transcription level. When transcriptome analysis of groups treated with genistein and control was performed, several genes that were significantly downregulated with genistein addition, thereby demonstrating their involvement in CIT production. A similar study by Huang et al. [117] investigated the effect of the addition of rutin and its derivatives, α-glucosylrutin and troxerutin, in fermentation media on *Monascus aurantiacus* Li AS3.4384 CIT production. The results showed that inhibition by rutin derivatives was significantly higher (>50%) than rutin (around 20%) when added at the same concentration. In addition, the media composition also affected the reduction in CIT yield. The highest reduction of about 90% was observed after 14 days of fermentation when 15.0 g/L of troxerutin was added to low-starch peptone containing liquid media [117].

In addition, several novel technologies have been incorporated in the food industry, such as high hydrostatic pressure (HHP), ultrasonication, and cold atmospheric pressure plasma (CAPP). Application of HHP on the infected olives successfully reduced the microbial population by 90–100% and degraded CIT up to 100%. Moreover, HHP also enhanced phenolic compounds and antioxidant activity [118]. The addition of 6–9% NaCl significantly reduced CIT production during olive storage [119]. Similarly, ultrasonication of red yeast rice degraded up to 87.7% of CIT produced by *Monascus purpureus* during fermentation [120]. On the other hand, CAPP degraded up to 50% of CIT developed by *Penicillium* sp. collected from wheat, oat, corn, and rice, without affecting the nutritional quality of grains [121].

## 9. Effects of Environmental Factors on Citrinin Production

Environmental factors such as temperature, pH, and light (especially during storage) affect the growth of fungus and the production of mycotoxin. Regulation of such environmental conditions, therefore, would help control the growth of fungus and thereby the release of toxins [122,123,124]. Wawrzyniak and Waśkiewicz [46] investigated the effect of temperature (10, 20, and 30 °C) and different cereal substrates (wheat, triticale, rye, barley, maize, rice) on the growth of *P. verrucosum* and the production of CIT while maintaining the moisture content. For the experiment, the cereals were moistened, autoclaved, and then inoculated with the fungal spores. The inoculated cereals were then kept under different storage temperatures for 40 days. To determine growth, ergosterol (ERG) was used as a biomarker and the mycotoxin content was determined using HPLC. The results showed maximum ERG at 30 °C, although growth was observed at all temperatures. Mycotoxin (CIT) was observed to be accumulated more at 20 °C in rice. The study suggested that irrespective of the temperature and cereal substrate, there is no strong correlation between the production of ERG and mycotoxin. This is reflected by the experiment since the optimal conditions for growth (30 °C) and CIT production (20 °C) do not coincide.

Another study by Yang et al. [125] investigated the effect of blue light on the yield of CIT. It was found that exposure to blue light for 15 min per day can significantly increase the toxin’s production in *Monascus purpureus*, and a decrease was observed when the exposure time was raised to 60 min/d. The addition of aminophylline and citric acid to the culture medium under illuminating conditions increased the expression of the mraox gene while decreasing the expression of lncRNA AOANCR, ultimately leading to a reduction in the production of CIT [125]. Furthermore, the effects of intensity of light and its color were investigated to determine the effect on biomass, pigment production, and CIT yield in *Monascus ruber* by Wang et al. [126]. The results showed a decrease in CIT accumulation when high-intensity blue light (1500 lx) was used. However, no significant effect was observed under low-intensity blue light (500 lx). The low intensity of green light (500 lx) was also studied for its impact and was found to increase CIT production via the upregulation of certain genes such as mrl1, mrl2, pksCT, and ctnA [126].

## 10. Detection Techniques

### 10.1. Sample Preparation

Sample preparation for the detection of CIT involves a process of extraction and clean-up that plays an important role in analyzing CIT with improved sensitivity, precision, accuracy, and specificity. Extraction is mainly carried out using solvents like acetonitrile and methanol in combination with other salts such as sodium chloride, potassium chloride and citric acid, and solvents like formic acid, acetic acid, and water. Some extraction processes even involve acidification using undiluted hydrochloric acid, sulfuric acid, or phosphoric acid to improve recovery and reproducibility [17]. Further, various clean-up methods have been reported for purification of samples containing CIT, for example liquid–liquid/solid extraction (LLE/LSE), dispersive liquid–liquid microextraction (DLLME), solid-phase extraction (SPE), immunoaffinity columns (IAC), and the quick, easy, cheap, effective, rugged, and safe method (QuEChERS) [127].

The LLE/LSE method for sample preparation is performed using polar organic solvents like ethanol, methanol, acetonitrile, and polyethylene glycol [128,129]. However, with this method, it is difficult to extract all analytes of interest with good recoveries, as they comprise of an extensive range of physicochemical properties. This method involves co-extraction of polar matrix components from the organic solvents used in the extraction process, thereby entailing a clean-up step. Thus, making the method unsafe due to the use of large amounts of toxic organic solvents, apart from making it time consuming and labor-intensive [130].

A fast and efficient extraction method DLLME uses small quantities of organic solvents in a three-component solvent system comprising a hydrophobic solvent (such as carbon tetrachloride), hydrophilic solvent (such as acetone), and an aqueous phase. In this method, the high surface area of hydrophobic solvent microdroplets expedites the extraction process followed by centrifugal separation of the analytes. However, the efficacy of this method for CIT sample preparation depends upon the nature of organic solvent being used, i.e., the ability to extract analytes of interest without significant contamination, lower solubility in aqueous samples, and compatibility with the instrument of analysis [131]. In SPE, the target analytes are retained in the solid media that are then recovered for analysis by solvent elution or thermal desorption. This method has several advantages over liquid extraction methods, for instance, higher selectivity and recovery, less solvent usage, and ease of online and offline automation. Commonly used sorbents that are used in the SPE method for the isolation of CIT include octadecylsiloxane-bonded silica sorbents (C_18_ phases), molecularly imprinted polymers (MIP), mixed-mode ion exchange resins, primary/secondary amines, and zirconia-coated silica [127].

Using the IAC method of sample preparation negates the effect of co-extraction of matrix interferences that is commonly found in liquid- and solid-phase extraction methods. This is done by immobilizing one of the interacting species (labelled as affinity ligand) on a solid support (for example, chitosan, silica, carbohydrate-related materials, sol–gel, and synthetic organic supports) through molecular recognition, followed by passing the sample over affinity sorbent. With this method, CIT is usually isolated using commercially available CitriTest IAC, which has improved sensitivity but accounts for strenuous and expensive production of antibodies [127]. However, using synthetic molecularly imprinted polymers can be an alternative, as they have better chemical and thermal stability along with higher extraction capacity [132]. Sample preparation using the QuEChERS method is carried out in two steps: (1) Extraction by establishing an equilibrium between the aqueous phase and organic phase based on salting-out; and (2) clean-up using dispersive solid-phase extraction (d-SPE). However, various studies have reported a modified QuEChERS method, where the clean-up is done by filtration [133]. Selective extraction of CIT has been reported to be carried out by using acetonitrile as a solvent due to its compatibility with the detection instrument (LC/MS) [127].

### 10.2. Detection and Quantification Methods

#### 10.2.1. Thin-Layer Chromatography (TLC)

TLC uses visual or fluorodensitometry procedures with a recovery limit of 0.01 ppm for quantitative as well as qualitative detection of mycotoxins, including the assessment of purity, separation, and the identification of organic compound heating [23]. In TLC, CIT separation is completed using various solvents, and the chemical validation of CIT is performed using two types of treatment approaches. The first involves saturation of TLC plate with acid-organic solution followed by exposing the TLC plate (with developed chromatogram) to the vapors of acetic anhydride/pyridine. The second treatment involves direct immersion of the TLC plate in an aluminum chloride reagent. These treatments transformed CIT into a new fluorescent compound that is detected under 365 nm light. However, with this method the fluorescence developed is weak and unstable. This yellow CIT fluorescence could be strengthened and sensitized and converted to blue by using another pre-treatment that involves aluminum chloride spray followed by heating [23]. CIT analysis using TLC has become limited, owing to its disadvantages of poor sensitivity and accuracy. Only Guo et al. [134] has recently studied the use of TLC for screening CIT in Liupao tea leaves inoculated with *Penicillium citrinum* strains.

#### 10.2.2. Colorimetric Technique of Detection

Under visual detection, the colorimetric technique is one of the most common methods for CIT detection. The conjugated planar structure imparts a natural fluorescence to CIT, that can be evaluated qualitatively and quantitively using a fluorometer. This natural fluorescence can be further intensified in acidic environments [23]. Apart from this, other ultrasensitive methods for visual detection of CIT in the nano molar range have also been reported. For instance, carbon dot is the most recently identified luminescence material used as a fluorescence probe for CIT detection in picomole range [135]. The authors here revealed that diammonium citrate and urea synthesized the nitrogen-doped aqueous soluble carbon dot (CD) emitted in the yellow region. This onsite CIT visual detection sensing platform statically quenched the yellow fluorescence of CD in the presence of Congo red dye as CIT has a higher binding preference with CD as compared to Congo red. Mg^2+^ further expedited the reversion of red fluorescence of CD-Congo red assembly to yellow in the presence of contaminated samples containing CIT. This method is supposed to work well even in the presence of other mycotoxins, which is a common occurrence in contaminated samples and is reproducible, sensitive, and cost-effective.

#### 10.2.3. High-Performance Liquid Chromatography (HPLC)

HPLC is one of the most common instrument-based techniques used to detect CIT in food and feed samples. It is usually combined with fluorescence, ultraviolet, and amperometric detection for high selectivity to achieve even low detection levels of CIT.

#### 10.2.4. Liquid Chromatography-Mass Spectroscopy (LC-MS)

LC-MS is a high throughput analytical method for CIT detection with reduced costs, labor, and time. LC-MS/MS systems with a triple quadrupole analyzer (QqQ) is the most widely reported system used for the determination of CIT in food and feed samples. Apart from this, recently a high-resolution LC-MS/MS system coupled with Qtrap mass analyzer was reported for the detection of CIT due to its full-scan mode operation that provides high specificity irrespective of the number of other mycotoxins detected [136]. Similarly, Li et al. [137] reported lower values of limit of detection (LOD) and limit of quantitation (LOQ) by the UHPLC-MS/MS method (as compared to the HPLC-FLD method) in Chinese liupao tea, which demonstrated higher sensitivity of this method in CIT detection. LC coupled to multiple MS possesses numerous advantages for CIT detection, including increased sensitivity and selectivity, adaptability to different types of sample preparation methods, compatibility with a wide range of sample matrices, rapid acquisition, and higher confidence in CIT identification, confirmation, and quantification along with other mycotoxins with a single method at regulated levels [127]. Tangni et al. [138] harmonized CIT determination in food and food supplements made up of red yeast rice, wheat flour, and *Ginkgo biloba* leaves, and determined their homogeneity and stability using the LC-MS/MS method. The analytical method proposed by the authors was found to be suitable and standardizable for citrinin determination at levels that could be contemplated by the European Commission.

#### 10.2.5. Liquid Chromatography Fluorescence Detection (LC-FLD)

The natural fluorescence of CIT can be an effective alternative for its detection. Since only a few mycotoxins possess this property, this method cannot be used for multi-mycotoxin analysis. Typical excitation and emission wavelengths for CIT fluorescence detection have been reported to be 330–335 nm and 497–500 nm, respectively [127]. However, there have been instances where CIT in foods like red yeast rice [139] and coffee samples [140] could not be detected using this method, implying lower sensitivity and selectivity for CIT detection.

#### 10.2.6. Liquid Chromatography UV/Visible Detection (LC-UV/Vis)

HPLC-UV/Vis is the least-reported method for CIT detection owing to its reduced selectivity and sensitivity than even fluorescence detection. However, since it is a simple and economical method, efforts can made to use this method in combination with a few pre-treatments for the estimation of CIT and other mycotoxins in food and feed.

#### 10.2.7. Enzyme-Linked Immunosorbent Assay (ELISA)

ELISA kits represent a portable and easy-to-apply in CIT detection technique that is commonly being used owing to its lower costs and fast analysis. In an ELISA assay, a complex is formed due to a competitive assay that exists between the analyte and a specific primary antibody or a conjugate of an enzyme. This complex then interacts with the chromogenic substrate to determine the amount of analyte present [141]. The CitriTest ELISA kit is a commercially available ELISA-based CIT detection method that has been used for the analysis of food and feeds for a decade [127]. Apart from this, food samples are also analyzed for CIT by employing polyclonal and monoclonal antibodies [142]. However, as compared to monoclonal antibodies, polyclonal antibodies are cheaper and easier to produce, with the advantage of possessing more binding places on the antigen [141]. However, due to the tendency of polyclonal antibodies to cause cross reactivity in ELISA detection, this method for CIT estimation is not preferred. Recently, a method of CIT detection using ELISA was modified to a competitive indirect ELISA (ciELISA) format for grain-based food samples that had high specificity for CIT without any cross-reactivity with other mycotoxins [142]. The assay was developed with CIT-BTG (bovine thyroglobulin), as it had the best antibody titer and inhibition. This assay was also found to be nine times more sensitive than conventional ELISA [137]. To further increase the sensitivity of this method for CIT detection, Huang et al. [143] used an environmentally friendly signal amplification strategy of immuno-polymerase chain reaction (IPCR) that broadened the linear range as compared to phage ELISA.

#### 10.2.8. Immunochromatographic Assay (ICA)

The properties of ICA make it a user-friendly method for CIT detection due to its suitability, simplicity, high speeds, and lower costs [144]. ICA assisted by molecularly imprinted biopolymers allows biological detection of multiple mycotoxins in contaminated agricultural products [145]. Commonly used molecularly imprinted biosensors using colloidal gold as a signal label have been found to possess insufficient sensitivity and cannot detect mycotoxins in lower concentrations. Thus, Xu et al. [146] improved the sensitivity of ICA by developing a dual fluorescent ICA (DF-ICA) using europium nanoparticles (EuNPs) due to its high fluorescence, long fluorescence lifetime, and non-toxic effects. The authors noted that the limits of detection (IC_50_) were 0.06 and 0.11 ng/mL and the average recovery for simultaneous determination of CIT was in the range of 86.3 to 111.6%. Similarly, a ratiometric detection strategy was introduced to improve the traditional molecularly imprinted electrochemical sensors that had limited practical applications due to their poor stability and reproducibility [147]. For CIT detection, Hu et al. [148] fabricated a sensor by electropolymerization with thionine as a monomer. Thionine and CIT anchorage were supported on the ample surface area provided by ionic liquid decorated boron and nitrogen co-doped hierarchical porous carbon (BN-HPC) as a supporter. With [Fe(CN)^6^]^3−/4−^ adopted as an indicating probe, a wide range of CIT detection was provided with a recovery of 97 to 110%.

#### 10.2.9. Capillary Zone Electrophoresis (CZE)

The CZE method for detection of mycotoxins was developed in response to the drawbacks that were observed for the instrument-based (HPLC and GC) and biological detection methods (TLC, ELISA, and paper-based colloidal gold testing). Drawbacks of instrument-based methods include high costs, complex pre-treatments, time-intensiveness, and the usage of large amounts of organic solvents, while the drawbacks of biological methods are possible false-positive results, difficult reproducibility, and sensitivity [149]. CZE uses differences in the charge to size ratio in the electric field that ionizes CIT at a basic pH due to the presence of carboxylic groups in it [127]. CZE along with an ultraviolet detector (CZE-UV) was used to detect CIT along with other mycotoxins in pepper, with a comparison with the results obtained by using the previously validated method of HPLC-FLD [150]. Satisfactory linearity was observed between the signal and CIT concentration (in the range 4.5 to 150 μg/kg) with an LOD range of 0.3 to 2.5 μg/kg. The authors revealed that the CZE-UV method was simple and quick, with higher analyte separation efficiency and sensitivity. In addition to this, CZE is a greener method as compared to LC, as it does not generate chemical waste [151].

## 11. Masked Mycotoxins as a Major Concern in Detection

The phenomena of growth of plants and fungi as well as the processing of foods bring about certain structural modifications in mycotoxins which lead to metabolites known as modified mycotoxins or masked mycotoxins. These modified mycotoxins can be either of the matrix-entrapment type (formed by physical dissolution or matric entrapment), or the chemically modified type (formed due to chemical or biological modifications) [152]. Some of the common modified toxins present in food crops like wheat, maize, and rice in substantial amounts include 3-acetyl-deoxynivalenol (3-acetyl-DON), deoxynivalenol-3-glucoside (DON-3-G), and zearalenone-14-glucoside (ZEN-14-G) [153,154]. Consumption of these modified mycotoxins poses potential risks to human health [90]. Since the physical matrices and chemical derivatives of masked mycotoxins cannot be detected by routine analytical methods, it is difficult to further evaluate their toxicological impacts, thus making them untraceable [155].

CIT analysis based on existing methods is exigent owing to its poor reproducibility and comparability [127]. Hou and co-authors have very recently used a modified method of UPLC-FLD by including a pre-treatment step of acid hydrolysis that increased the CIT content in *Hongqu* significantly from 35.28% to 458.13% in CIT positive samples, thereby not only increasing the efficiency of fluorescence detection method but also unmasking the modified CIT that was believed to be matrix-associated (physically dissolved or trapped in the matrix compounds) in the contaminated food samples [155]. Thus, it can be implied that modified CIT (as a mycotoxin of concern) can be quantified using an appropriate combination of pre-treatment methods such as transformations using a hydrolytic process involving alkaline, acidic, and/or enzymatic methods [156,157]. In addition, integrated strategies of the analysis of masked forms mycotoxins, as suggested by Lu, et al. [158], could be applied for masked CIT to ensure proper detection and ultimately the safety and security of food and feed.

## 12. Degradation Kinetics

CIT contamination in food and feed can cause economic losses as well as concerns for human and animal health [159]. Therefore, suitable degradation and management strategies are crucial [160]. Different physical, chemical, and biological approaches are evaluated for the degradation and management of CIT at in-vivo and in-vitro conditions (Table 3). For physical methods, high-temperature treatment is the most common step during cooking for various purposes such as softening, taste improvement, degradation of toxic compounds, and sterilization. CIT is considered heat unstable and various chemical changes have been reported due to heat treatment at different conditions. Trivedi et al. [161] noted the partial degradation of CIT into less cytotoxic forms when heated with water above 120 °C. Further, boiling of CIT aqueous solution at 100 and 140 °C gradually degraded CIT into less toxic compounds, namely CIT-H1 and CIT-H2 [162]. The presence of moisture easily detoxified CIT at 140 °C, while the higher temperature was required to decompose at dry conditions. CIT degradation started at 160 °C and was fully decomposed at 170 °C [112]. Dry heat did not cause any change in the CIT concentration of fermented red mold rice; however, heating in aqueous condition initially increased the CIT level, but with a further increase in temperature above 175 °C, the CIT level decreased [163]. Besides heat, CIT is also light-sensitive. The application of white and blue light wavelengths completely degraded CIT content in in vivo conditions [164] and reduced about 79% of CIT during fermentation and *Monascus* production [165].

Similarly, the chemical method employs various compounds for degradation purposes. A mixed solvent made up of phosphate–ethanol was reported to remove 91.6% CIT content within 70 min from *Monascus* species-fermented red mold rice [163]. The addition of medium-chain fatty acids in the *Monascus ruber* culture medium inhibited the CIT production by the formation of hydrogen peroxide, and the pigment production was increased up to 50% [166]. Some flavonoids such as rutin, α-glucosylrutin, or troxerutin were applied to the fermentation process which was initiated by *Monascus aurantiacus* Li AS3.4384. The addition of an equal amount (5.0 g/L) of rutin, α-glucosylrutin, or troxerutin degraded CIT content by 29.2%, 54.7%, and 40.6%, respectively. However, the CIT inhibition efficacy of troxerutin was between 53.7 and 87.9% at the end of the experiment, where the factor responsible for variation in CIT inhibition efficiency was the concentration of troxerutin and fermentation medium [117]. Besides this, absorbers are being used to tackle mycotoxins. For example, activated charcoal was found effective to ameliorate the toxic effect of mycotoxins including CIT when it was fed to broiler with feed. However, the combination of activated charcoal (0.4%) and lyophilized yeast (0.2%) culture exhibited higher efficacy in mitigation of mycotoxins [167]. An aqueous extract of mint inhibited up to 73% of the CIT produced from *Penicillium citrinum*, but did not inhibit fungal growth and the CIT reduction efficiency was concentration-dependent [168].

Hence, due to several limitations of physical and chemical methods, various biological methods as alternatives have been examined for CIT degradation. Several microorganisms can degrade CIT. CIT is nephrotoxic, causes damage to genetic material, and alters the differentially expressed proteins. However, proteomes and transcriptomes of *Cryptococcus podzolicus* Y3, when treated with 10 μg/mL CIT, exhibited defense responses and drug metabolism, and consequently degraded CIT [160]. The degradation of CIT by *C. podzolicus* Y3 is due to its intracellular enzyme(s), not by uptake or adsorption of CIT. The degradation of CIT is dependent on various factors such as time, temperature, concertation of cells, and pH [169]. In in vivo conditions, *Rhodotorula mucilaginosa* significantly (93.10%) degraded CIT produced by *P. digitatum* in the 48 h of the experiment [170]. *Aspergillus*, *Cladosporium*, and *Penicillium* are the major genera that infect the paddy rice and produce CIT during storage. The addition of *Trichoderma hamatum* in in vivo conditions significantly reduced the population of *Penicillium viridicatum* and inhibited CIT content [171]. Further, *Klebsiella pneumoniae* strain NPUST-B11 isolated from soil sample was effective in degrading CIT [172]. In addition, Kanpiengjai et al. [173] collected 96 bacterial strains from various sources in which *Rhizobium borbori* PS45 and *E. cloacae* PS21 were found to be effective for CIT degradation. However, the efficacy of microorganisms for CIT degradation depends on the concentration, pH, temperature, and substrate used [170].

## 13. Management and Control Strategies

CIT is mainly produced by members of the genus *Aspergillus* and *Penicillium*. Fruits or grains may get infected by these microorganisms at any stage: pre-harvest (cultivation, cultural operations), during harvest, and post-harvest (handling, processing, or storage) conditions. These can be managed at the field level by adopting good agricultural practices (GAPs), good manufacturing practices (GMPs), and good storage practices (GSPs) such as the selection of resistant cultivars/varieties, nutrient management, floor management, clean cultivation, proper weeding and tillage operations, the application of optimum level of plant protection chemicals, and harvesting at the proper maturity stage. After harvest, proper handling, adequate moisture, and suitable storage conditions are helpful to mitigate mycotoxins [177,178,179,180,181,182] Ostry et al. [133] reported the natural occurrence of CIT in grapes. They identified 23 strains of *Penicillium expansum* in 25 samples of grapes. Some of them were responsible for the production of CIT. The authors recommended proper monitoring of fungal colonization in the field and during harvesting because mycotoxins make fresh produce unfit for consumption and may not be degraded properly during processing. For their management, some chemical and biological approaches can be adopted, as discussed in the earlier section.

Commercial fungicides such as Aliette, Rovral, Cantus, Ortiva, Luna Experience, Fenomenal, and Mancozeb are beneficial to inhibit the growth of filamentous fungi at the pre-harvest stage. However, sometimes plant protection chemicals can induce the formation of mycotoxins [183]. Postharvest benzo-(1,2,3)-thiadiazole-7-carbothioic acid S-methyl ester (BTH) treatment of peach prevents the infection of *Penicillium expansum* [184]. However, some fungi develop resistance against fungicides, thereby raising concerns [185]. Hence there is a need for better alternatives.

Harvesting at the right maturity stage and proper handling reduce the bruising injury. Similarly, proper grading for selecting good quality products by culling damaged and overripe products can mitigate the chance of microbial contamination. Further, postharvest contamination of fungal infection can be successfully inhibited using plant extracts such as jasmonates [186]; cinnamon bark extract [186]; pomegranate peel extract [187]; essential oils from *Citrus aurantium* [188]; clove oil [189]; the application of irradiation [190,191,192,193]; hot water treatment (HWT) [194]; salts [195,196]; mixed treatments such as the use of sodium salts with HWT [197]; salts and wax [198]; the use of biocontrol agents [199,200], and the combination of HWT and *Debaryomyces hansenii* [201]. In the post-harvest phase, a proper storage facility, cleanliness, and the right moisture level are also crucial. Dust and left out stored material may act as an inoculum and can be a source of infection in the subsequent storage materials. Tangni and Pussemier [3] reported that the dust contains about 137–344 ng/g CIT and the presence of dust material in storage may cause CIT contamination in stored wheat grain. The mycotoxicity depends on the water activity of grain and the mycotoxigenic potential of inoculants. Moisture condensation and migration were observed in wheat grain even when stored below the threshold of 14.5% moisture. Furthermore, the nature of the condensation affected *P. verrucosum* contamination and CIT formation [79]. Application of ozone in stored food grain inhibits the growth of *Aspergillus flavus* and *Penicillium citrinum* and the formation of mycotoxin [175]. CIT contamination and concentration can also be managed during processing by adopting the principle of HACCP. The selection of suitable microbial strains and alterations of suitable substrates for fermentation significantly inhibit CIT concentration in the end product. Marič et al. [8] found that the ‘EBY-3’ strain of *Monascus purpureus* produced the highest pigment yield and inhibited CIT formation after 21 days of fermentation process when rice was used as substrate. Furthermore, the application of some natural products like mint extract [168] and neem leaf extract [202] have been reported to inhibit CIT production. The use of *Zataria multiflora* Boiss essential oil inhibited the growth of *P. citrinum* in lab conditions and CIT production in cheese [203,204]. A similar finding was also reported with the application of eugenol and thymol in Spanish cheese [205].

## 14. Conclusions

Consumption of citrinin-contaminated food and feed by both humans and animals has led to serious health concerns across the globe. The toxin could enter the food chains by contaminating the food and feed at any stage of agricultural practice and in pre/post-harvest conditions. Due to the nephrotoxic and genotoxic nature of CIT, the health of both humans and animals is at greater risk. Therefore, proper Hazard Analysis Critical Control Point (HACCP) plans, Good Agricultural Practices (GAPs), and Good Manufacturing Practice (GMPs) could be effective in controlling the toxins during various agriculture and processing stages. In addition, various physical, chemical, and biological methods could be implemented to degrade and mitigate CIT production and contamination, thereby preventing its entry into the food chain. Further, the toxin is reported to degrade into various forms and exists in masked forms. Hence, rapid and precise detection methods become essential as well as challenging in terms of their identification, quantification, and mitigation. This highlights the necessity of concise and reliable detection methods for their management. Limited information is available on the masked forms of CIT in food and feed, and these forms are likely to remain undetected and under-reported, thereby presenting a hidden threat to food safety and security. Hence, future research should emphasize an in-depth investigation of the masked forms of CIT to obtain easy, rapid, and precise detection and mitigation strategies.

## Figures and Tables

**Table 1:** Major citrinin producers among fungal species in foodstuffs.

Genera	Subgenus	Series	Species
*Penicillium*	*Furcatum*	-	*P. citrinum* Thom
	*Penicillium*	Expansa	*P. expansum* Link
	*Penicillium*	Corymbifera	*P. radicicola* Overy & Frisvad
	*Penicillium*	Verrucosa	*P. verrucosum* Dierckx
	*Penicillium*	-	*P. viridicatum* Westling
	*Penicillium*	-	*P. camemberti* Sopp
*Aspergillus*	-	-	*A. carneus* Tiegh
	-	-	*A. niveus* Blochwitz
	-	-	*A. oryzae*
	*Circumdati*	-	*A. terreus* Thom
*Monascus*	-	-	*M. purpureus* Went
	-	-	*M. ruber* Tiegh

**Table 2:** Occurrence of citrinin in food and feed around the world.

Food Matrix	Country	Range (μg/kg)	Detection Technique	References
Amaranth	Spain	1.8–5.9	QuEChERS	[31]
Apples	Portugal	320–920	SPE-HPLC	[32]
	Portugal	3.06–5.37	TLC	[33]
	China	1.7–16.3	UPLC-MS/MS	[34]
	Croatia	240	TLC	[35]
Almond	Spain	3.0–7.4	UHPLC-MS/MS	[36]
	India	2.80–18.20	ELISA	[37]
Barley	Czech Republic	93.64	HPLC	[38]
Black Pepper	India	17.8	LC-MS/MS	[16]
Black olives	Turkey	350	TLC	[39]
	Morocco	0.2–0.5	HPLC	[40]
Breakfast cereals	France	1.5–42	HPLC-FD	[41]
	France	0.5–1.5	HPLC	[42]
Brown rice	Spain	6.4–10	QuEChERS	[31]
Buckwheat	Spain	1.5–6.9	QuEChERS	[31]
	Spain	0.62	LC-MS/MS	[43]
Cashew	India	4.70–9.80	ELISA	[37]
Cajna salami	Croatia	<1.0–1.0	HPLC	[44]
Cereals	Belgium	14.3	UHPLC-MS/MS	[45]
	Croatia	19.63	HPLC-FD	[41]
Cereal solid substrates	Poland	5.7–74.8	HPLC-FLD	[46]
Cereals and derivatives	Germany	<1–2.7	HPLC-FD	[41]
Cocoa	Belgium	3.4	UHPLC-MS/MS	[45]
Coriander	India	23.0	LC-MS/MS	[16]
Commercial beers	South Africa	6	TLC	[47]
Cumin	India	14.7	LC-MS/MS	[16]
Dried grape	Turkey	5.56	HPLC-FD	[48]
Dried white mulberry	Turkey	4.26–5.29	HPLC-FD	[48]
Dry ginger	India	19.4	LC-MS/MS	[16]
Family Cereal	Nigeria	1.2–151	LC-MS/MS	[49]
Fermented dry meat products	Croatia	<1.0–1.3	ELISA	[44]
Fenugreek	India	17.2	LC-MS/MS	[16]
Fruits	China	0.06–0.10	QuEChERS-HPLC-FLD	[50]
Grape	China	0.16	USAE-DLLME-HPLC-FLD	[50]
Ground rice	China	5–100	HPLC-DAD	[51]
Hazelnut	Spain	3.1–8.0	UHPLC-MS/MS	[36]
Industrially-processed complementary foods	Nigeria	1.2–151	LC-MS/MS	[49]
Infant formula	Nigeria	3.6	LC-MS/MS	[49]
Koji rice	USA	50–1000	IAC-HPLC	[52]
Lager beer	Czech Republic	0.2–10	SPE-HPLC	[32]
Liquorice root	Turkey	14.66–19.14	HPLC-FD	[48]
Monascus pigment powder	China	122–594	RP-HPLC	[53]
Maize	Serbia	5–547	LC-MS/MS	[54]
	China	4.71–18.49	ic-ELISA	[55]
	Mozambique/Burkina Faso	531–5074	LC-MS/MS	[56]
Macadamia nut	Spain	3.3–7.3	UHPLC-MS/MS	[36]
Medicinal and aromatic herbs	Spain	16.5	ELISA	[57]
Mushroom	USA	400	IAC-HPLC	[52]
Ogi	Nigeria	0.8–159	LC-MS/MS	[49]
Olive	China	0.05	IAC-HPLC-FLD	[50]
Orange	China	40.3	UPLC-MS/MS	[34]
Parboiled rice	India	12–55	HPLC	[58]
Pear	China	0.16	USAE-DLLME-HPLC-FLD	[50]
Peanut	Spain	2.9–8.9	UHPLC-MS/MS	[36]
Pine nuts	Spain	5.5–9.0	UHPLC-MS/MS	[36]
Pumpkin seed	Spain	2.6–7.3	UHPLC-MS/MS	[36]
Pistachio	Spain	4.4–8.5	UHPLC-MS/MS	[36]
	India	4.57–15.80	ELISA	[37]
Quinoa	Spain	5.3–6.9	QuEChERS	[31]
Raisin	India	2.84–17.40	ELISA	[37]
Red chilli	India	12.5	LC-MS/MS	[16]
Red rice	Spain	2.8–6.2	QuEChERS	[31]
	Malaysia	0.23–20.65	ELISA	[59]
Red kojic rice	China	50	HPLC-FD	[60]
	Japan	200	MFEI	[61]
	China	100	IAC	[62]
Red mold rice	USA	50–2500	IAC-HPLC	[52]
	Malaysia	0.23–20.65	HPLC	[59]
	USA	24–189	HPLC-UV	[63]
	Taiwan	5742–27,000	HPLC-FLD	[64]
	China	49–13,550	HPLC-FLD	[64]
	China	7.5–120	HPLC	[64]
Red fermented rice	China	140–44,240	LC-MS/MS	[65]
	China	0.12–5.71	HPLC	[66]
	Croatia	95–98	Rapid LC/DAD/FLD/MS	[67]
	China	0.14–44.24	LC-MS/MS	[65]
	China	250–825	HPLC-FLD	[65]
Red yeast rice	China	2.33–32.47	MFCI	[68]
	Belgium	3.6–121,097	UHPLC-MS/MS	[45]
	China	57.28	HPLC-FLD	[69]
	China	100.6–443.6	IAC-HPLC	[70]
	China	16.6–5253	LC-MS/MS	[68]
	Croatia	98	LC-MS	[71]
Red yeast rice powder	China	0.10–5.41	RP-HPLC	[53]
Red yeast powder	China	55	HPLC-FD	[62]
Red yeast rice food additives	China	127–4960	LC-MS/MS	[68]
Red yeast rice functional food and medicine products	China	16.6–62.5	LC-MS/MS	[68]
Rice	Argentina	0.5–50	ELISA	[61]
	Vietnam	0.42	HPLC-FLD	[72]
	Iran	5–21.05	LC-MS/MS	[73]
	Vietnam	0.38–0.42	UHPLC-FL	[74]
	China	0.11	LLE-HPLC-FLD	[50]
	China	0.7–1.0	SPME-LC-FLD	[50]
	Spain	5–200	HPLC-DAD	[75]
	Japan	49–92	HPLC	[76]
	Canada	700–1130	HPLC	[76]
	China	9.65–19.85	ic-ELISA	[55]
	Iran	5–21.05	HPLC	[58]
	India	49–92	HPLC	[58]
Sausages	Croatia	<1.0–1.0	ELISA	[44]
Semi-dry sausages	Croatia	<1.0	HPLC	[44]
	Croatia	<1.0	ELISA	[44]
Spices	Belgium	1.4–19.8	UHPLC-MS/MS	[45]
Spelt	Spain	2.6–10.4	QuEChERS	[31]
Soybean	Egypt	270	HPLC	[77]
Sunflower seed	Spain	4.6–10.2	UHPLC-MS/MS	[36]
Sweet cherries	China	2.2–7.9	UPLC-MS/MS	[34]
Tomato	China	1.1–8.4	UPLC-MS/MS	[34]
Tom bran	Nigeria	1.7–1173	LC-MS/MS	[49]
Tom bran	Nigeria	0.8–1173	LC-MS/MS	[49]
Walnut	Spain	4.6–7.7	UHPLC-MS/MS	[36]
White rice	Spain	4.0–6.4	UHPLC-MS/MS	[31]
Wheat	Tunisia	0.1–170	HPLC	[78]
	Canada	175.2	HPLC	[79]
	China	4.77–19.49	ic-ELISA	[55]
	Czech Republic	0.09	HPLC-FD	[80]
Wheat flour	Belgium	0.1	UHPLC-MS/MS	[45]
	Czech Republic	19.2–2068.6	HPLC-FD	[80]
Winter salami	Croatia	<1.0–1.3	HPLC	[44]
Feed				
Feed	Burkina Faso	341	LC-MS/MS	[56]
Complete animal feeds	Belgium	1.9–2.0	UHPLC-MS/MS	[45]
Maize silage	France	1.5–5.0	LC-MS	[81]
Maize silage	France	5–25	LC-MS	[82]
Maize silage	France	2–1.5	LC-MS	[83]
Compounded feeds	Russia	10–182	ELISA	[84]
Maize gluten	Russia	62	ELISA	[84]
Wheat bran	Russia	397	ELISA	[84]
Soy-bean oilseed meal	Russia	30	ELISA	[84]

UHPLC-MS/MS: Ultra-high-performance liquid chromatography tandem mass spectrometry; UHPLC-FL: Ultra-high-performance liquid chromatography and fluorescence detection; LLE-HPLC-FD: Liquid–liquid extraction–high performance liquid chromatography–fluorescence detector; IAC-HPLC-FD: Immunoaffinity column–high performance liquid chromatography–fluorescence detector; SPE-HPLC-FD: Solid phase microextraction–high performance liquid chromatography–fluorescence detector; QuEChERS: quick, easy cheap, effective, rugged, and safe high performance liquid chromatography–fluorescence detector; USAE-DLLME-HPLC-FLD: ultrasound solvent extraction–dispersive liquid–liquid microextraction–high performance liquid chromatography–fluorescence detector; SPE-HPLC: Solid-phase extraction–high performance liquid chromatography; MFEI: micro fluidic electrochemical immunosensor; icELISA: indirect competitive enzyme-linked immunosorbent assay; UHPLC-MS/MS: Ultra-high performance liquid chromatography coupled with tandem mass spectrometry: RP-HPLC: Reversed-phase HPLC; MFCI: microsphere-based flow cytometric immunoassay; IAC: Immunoaffinity columns; USAE-DLLME: ultrasound-assisted extraction combined with dispersive liquid–liquid microextraction.

**Table 3:** Various degradation methods for controlling citrinin in food and feed.

Degradation Methods	Experimental Details	Key Findings	References
Physical			
Light (Blue light)	*Monascus* production	Decreased CIT by 79%; 28.5% increase in pigment production	[165]
Blue light	In vivo	Blue light completely degraded the CIT	[164]
Temperature/Heat	Heating under aquous condition Temperature: 90–130 °C Time: 10–20 min	Partial degradation and formation of low cytotoxic substances; increase in temperature and time above 120 °C to form another less cytotoxic substance	[161]
Heating/boiling	Heating at 100–140 °C in aqueous medium	High-temperature treatment degraded CIT into CIT H1 and H2	[162]
High hydrostatic pressure (HHP)	Time: 5 min Pressure: 250 MPa Temperature: 35 ± 1 °C	90–100% of the microbial population was reduced; the CIT level was reduced up to 100%; increased phenolic compounds; enhanced antioxidant activity	[118]
Cold atmospheric pressure plasma	Power output: 50 kV, 100 watts Electron frequency: 30 kHz Gas flow: 6 L/min	Reduced 50% of CIT; no negative effect on nutrients	[121]
Magnetic nanoparticles		Formation of a CIT–nanoparticle complex; effective in CIT removal; can be used in the food industry; is difficult to operate on a large scale	[174]
Ultrasonication	Power: 250 W Liquid: solid ratio 40:1 Time: 50.7 min, temperature: 20 °C	Removed up to 87.7% CIT from red yeast rice	[120]
Chemical			
Ozone	O_3_ treatment: (40 and 60 μmol/mol Time: 180 min	CIT level reduced from 173.51 μg/kg to 42.90 μg/kg 180 min after treatment	[175]
Medium-chain fatty acids	In vivo *Monascus ruber*	Improved pigment formation; reduced CIT production in the process	[166]
Flavanoids	*Monascus aurantiacus* Li AS3.4384	Inhibition of CIT formation up to 87.9%	[117]
*Monascus* species-fermented red mold rice	45% ethanol, 1.5% phosphate, and extraction for 70 min	Reduced CIT level by 91.6%; maintained 79.5% monacolin K	[163]
Biological			
Genistein	*Monascus* mold (used to produce *Monascus* pigments, monacolin K, and ergosterol)	Suppressed acetyl- CoA formation; reduced CIT content; reduced significant differential metabolites	[176]
*Cryptococcus podzolicus* Y-3 cells	-	In response to CIT stress, DNA repair, antioxidative activity, and the TCA cycle were activated; degradation of CIT	[160]
*Cryptococcuspodzolicus* Y3	-	Degradation up to 98%; intracellular enzyme caused degradation; degradation into less toxic compounds; degradation was factor-dependent	[169]
*Rhodotorula mucilaginosa*	-	Degradaded CIT by 91.67% at pH 4.0 and 28 °C; degradation was factor-dependent	[170]
*Klebsiella pneumoniae* strain NPUST-B11	-	Ful degradation of CIT after 10 h of incubation.	[172]
*Rhizobium borborid*	Temperature: 30 °C Time: 120 h	*R. borbori* PS45 and *E. cloacae* PS21 were found to be the most promising among the collected strains; they caused 63.4% and 43.6% reduction, respectively	[173]
Adsorbents	Activated charcoal and 0.4% lyophilized yeast culture (0.2%) with feed	Ameliorated toxic effect of mycotoxin to broilers	[167]

## Data Availability

Not applicable.
